# Elucidating the Three‐Dimensional Structure of Piracetam through Rotational Spectroscopy

**DOI:** 10.1002/open.202400490

**Published:** 2025-03-15

**Authors:** S. Mato, S. Municio, J. L. Alonso, E. R. Alonso, I. León

**Affiliations:** ^1^ Química Física—Química Inorgánica Department Universidad de Valladolid Grupo de Espectroscopia Molecular (GEM) Edificio Quifima Laboratorios de Espectroscopia y Bioespectroscopia Parque Científico Universidad de Valladolid 47011 Valladolid Spain

**Keywords:** piracetam, FTMW spectroscopy, non-covalent interactions, drugs, conformers

## Abstract

Herein we report on the most stable structures adopted by piracetam, a nootropic drug, in isolated conditions. A chirped pulse Fourier transform microwave spectrometer (CP‐FTMW) coupled with a laser ablation source has been employed to explore the broadband rotational spectrum of piracetam in the 6.0–14.0 GHz range. Two conformers have been observed. The most stable conformer of piracetam adopts an *exo* configuration of the ring and is mainly stabilized through a N−H⋅⋅⋅⋅⋅⋅O=C hydrogen bond between the amide group and the rings′ carbonyl oxygen. The second conformer is close in stability and only differs in the *endo* disposition of the ring. The results show a low interconversion barrier between both conformers.

## Graphical abstract

Neutral molecules of the nootropic drug piracetam are generated by laser ablation and structurally characterized by microwave spectroscopy. Two conformers differing in the *exo*/*endo* configuration of the pyrrolidine ring are detected, with *exo* being the predominant form.

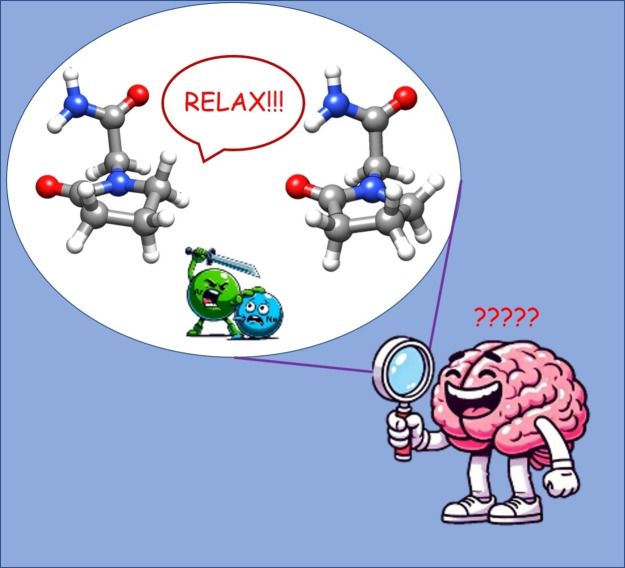

## Introduction

**Figure 1:**
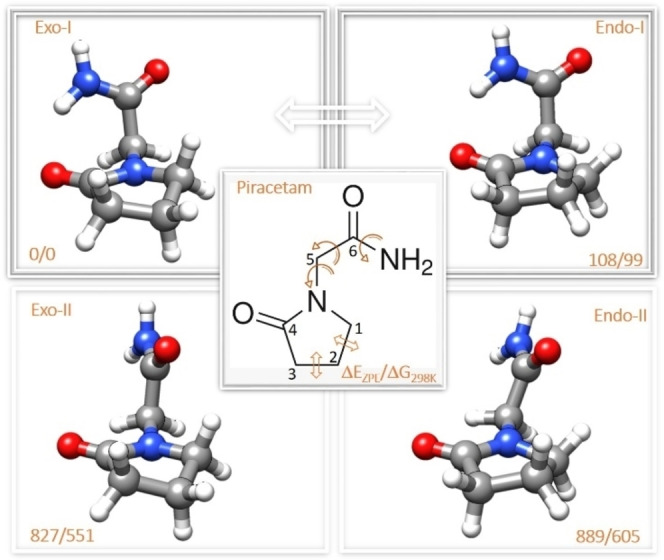
The four stable conformers of the piracetam molecule. The middle panel shows the structure of piracetam with the arrows indicating the torsional degrees of freedom and the two possible orientations of the ring: *exo* and *endo*. The top name shows the ring configuration (*exo*/*endo*) and the stability order within such configuration (I or II). The bottom numbers are the energy difference considering the zero‐point energy correction (ΔE_ZPE_), as well as the entropic difference at room temperature and 1 bar (ΔG). The white arrow indicates a low barrier separating both ring configurations (see main text).

Piracetam (2‐oxo‐1‐pyrrolidineacetamide, Figure 1) stands as a pioneering nootropic drug that has revolutionized the field of cognitive enhancement pharmaceuticals since its development in the 1960s.^[1]^ This synthetic derivative of the neurotransmitter γ‐aminobutyric acid (GABA) represents the prototypical member of the racetam family, characterized by its distinctive 2‐pyrrolidone nucleus. Despite its relatively simple molecular structure, piracetam exhibits remarkable pharmacological properties, including enhancement of cognitive function, memory formation, and learning capacity.[^2^, ^3^, ^4^, ^5^]

The molecule‘s unique mechanism of action involves the modulation of various neurotransmitter systems and the enhancement of neuronal membrane fluidity, leading to improved cellular communication and synaptic plasticity. Its ability to cross the blood‐brain barrier efficiently, coupled with its low toxicity profile, has established piracetam as a significant therapeutic agent in treating cognitive impairment and neurological disorders.[^4^, ^6^]

Research interest in piracetam continues to grow, driven by its potential applications in treating conditions ranging from age‐related cognitive decline to post‐stroke recovery. Understanding its molecular behavior, pharmacokinetics, and structure‐activity relationships remains crucial for developing more effective cognitive enhancers and expanding our knowledge of brain function and neuroplasticity. Therefore, the structural characterization of piracetam is essential, not only for quality control in pharmaceutical manufacturing but also for comprehending its physicochemical properties and derived properties. Due to the importance of its structural characterization, piracetam and its derivatives have been investigated by several authors using different techniques and in different phases. In the solid phase, two piracetam polymorphic crystal forms were initially observed,^[7]^ although later, five distinct polymorphs were reported.^[8]^ Two of these structures are stable under ambient conditions, as shown by X‐ray diffraction, Raman and near‐infrared spectroscopy studies of piracetam for quantifying polymorphic mixtures.^[9]^

In solution or solid state, the structures are the result, not only of the number and type of interactions in their building blocks but also with the surrounding media. Therefore, gas phase experiments provide a unique and ideal medium to study, understand, and model the interactions between small molecules free from any external interaction.[^10^, ^11^, ^12^, ^13^] There is a single experimental study in the gas phase, where the structure and conformations of piracetam was explored using electron diffraction and quantum chemical calculations.^[14]^ Only one structure was detected stabilized by the intramolecular hydrogen bond between the amine and the ring carbonyl group through an N−H⋅⋅⋅O=C hydrogen bond. Finally, a DFT computational study of piracetam was also done^[15]^ for the detected conformer in ref. [14]

One of the most powerful structural tools in physical chemistry is rotational spectroscopy. Since the frequencies of a given species with non‐zero dipolar moment are determined by the three moments of inertia in the principal inertial axes, the assignment, together with the selection rules, gives precise structural information. In this paper we will show that there are two conformers of piracetam in isolated conditions, and we will report the structure and rotational constants of the two conformers.

## Results and Discussion

### The Conformational Panorama of Piracetam

In a first step, we began the study with a comprehensive analysis of the most stable conformational isomers of piracetam. The first step involved scanning the Potential Energy Surface (PES) using molecular mechanics (MM) methodology. The MMFFs force field^[16]^ was used with an energy threshold of 50 kJ/mol and with no solvent. A total of 10 structures were obtained across all force fields. Due to their low precision, the energy values and spectroscopic parameters of the structures obtained through MM are inaccurate. Therefore, the obtained structures were re‐optimized using DFT calculations with the B3LYP‐GD3 functional[^17^, ^18^, ^19^, ^20^] and the 6‐311++G(d,p) basis set.^[21]^ The structures were also optimized using MP2^[22]^ with the same basis set. Of those initial structures obtained by MM, most were duplicated or converged to the same one, resulting in only four distinct conformers. The structure of these conformers is shown in Figure 1, labeled according to their relative stability and the configuration of the ring, i. e., *Exo‐I*, *Endo‐I*, *Exo‐II*, and *Endo‐II* conformers. The most relevant spectroscopic parameters are listed in Table 1, and the Cartesian coordinates can be found in Tables S01–S04 of the Electronic Supporting Information (ESI).

**Table 1:** Experimental spectroscopic parameters obtained for the two detected rotamers of piracetam compared with those calculated using B3LYP‐GD3/6‐311++G(d,p) and MP2/6‐311++G(d,p) for the four lowest energy conformers.

	Experimental		Calculated (B3LYP‐GD3/MP2)			
Parameters	Rotamer I	Rotamer II	Exo‐I	Endo‐I	Exo‐II	Endo‐II
*A* ^[a]^	2340.53783(173)^[h]^	2361.090(114)	2353/2279	2349/2316	2510/2543	2465/2451
*B*	1006.68679(112)	979.97168(267)	995/1036	980/997	921/963	908/928
*C*	809.00161(107)	790.24504(247)	796/820	787/794	797/852	748/786
*μ_a_*	Observed	Observed	3.3/2.9	3.6/3.4	1.2/0.7	1.4/1.5
*μ_b_*	Observed	Not Observed	0.9/0.9	1.0/1.1	2.1/2.1	1.1/1.4
*μ_c_*	Observed	Not Observed	0.5/0.4	0.1/0.0	2.8/2.2	2.8/2.8
*χ_aa_ (N_1_)*	1.078(287)	–	1.24/1.07	1.60/1.48	1.63/1.52	1.81/1.78
*χ_bb_ (N_1_)*	1.704(38)	–	1.87/1.68	1.98/1.74	2.01/1.78	1.68/1.52
*χ_cc_ (N_1_)*	−2.782(38)	–	−3.10/−2.75	−3.58/−3.22	−3.64/−3.30	−3.59/−3.30
*χ_aa_ (N_2_)*	−0.789(67)	–	−0.60/−1.04	−0.48/−0.68	1.88/1.44	1.83/1.75
*χ_bb_ (N_2_)*	1.030(105)	–	1.24/1.20	1.17/1.10	−2.44/−2.68	−0.03/−1.00
*χ_cc_ (N_2_)*	−0.242(105)	–	−0.64/−0.16	−0.70/−0.42	0.55/1.24	−1.80/−0.75
*ΔJ* ^[b]^	−0.1580(119)	–	–	–	–	–
*N* ^[c]^	121	19	–	–	–	–
*σ* ^[d]^	33.2	40.4	–	–	–	–
*ΔE* ^[e]^	–	–	0/0	109/124	998/691	1084/1006
*ΔE_ZPE_* ^[f]^	–	–	0/0	108/134	827/590	889/863
*ΔG_298K_* ^[g]^	–	–	0/0	99/135	551/384	605/565

[a] *A*, *B*, and *C* represent the rotational constants (in MHz); μ_a_, μ_b_, and μ_c_ are the values of electric dipole moment components (in Debyes); χ_aa_, χ_bb_, and χ_cc_ are the diagonal elements of the ^14^N nuclear quadrupole coupling tensor (in MHz); [b] ΔJ is the quartic centrifugal distortion constants (in MHz); [c] Number of measured hyperfine components/transitions. [d] RMS deviation of the fit (in kHz). [e] Relative energies (in cm^−1^) with respect to the global minimum. [f] Relative energies (in cm^−1^) respect the global minimum, considering the zero‐point energy (ZPE). [g] Gibbs energies (in cm^−1^) calculated at 298 K and 1 bar. [h]Standard error in parentheses in units of the last digit.

Thanks to the acquisition of these structures, we can draw some conclusions regarding the stability of each one. In the main text, we use B3LYP/6‐311++G(d,p) as a reference, but the results using MP2/6‐311++G(d,p) are similar. In the two most stable structures *Exo‐I* and *Endo‐I*, the structures are mainly stabilized through an intramolecular N−H⋅⋅⋅O=C hydrogen bond between the amide group and the carbonyl oxygen of the ring. As shown in Figure 1, the difference between the two most stable structures is the position of the ring, either in the *endo* or *exo* position. The *exo* position is slightly more stable than the *endo*, possibly due to the proximity of the CH_2_ group at the gamma position (C_γ_ or C_1_ in the figure) of the ring and the amide carbonyl, stabilizing this configuration about 100 cm^−1^.

The other two structures, *Exo‐II* and *Endo‐II*, sacrifice the N−H⋅⋅⋅O=C hydrogen bond present in *Exo‐I* and *Endo‐I* and are stabilized by an intramolecular C−H⋅⋅⋅O=C non‐covalent interaction between the methyl group of the ring and the amide carbonyl group. However, since a moderately strong hydrogen bond is lost, the structures are destabilized more than 800 cm^−1^. Again, the difference between *Exo‐II* and *Endo‐II* structures lies in the *endo*/*exo* configuration of the ring.

In summary, the calculations predict two stable conformers of piracetam, *Exo‐I*, and *Endo‐I* conformers, that should be populated enough to be detected.

### Rotational Spectrum of Piracetam

**Figure 2:**
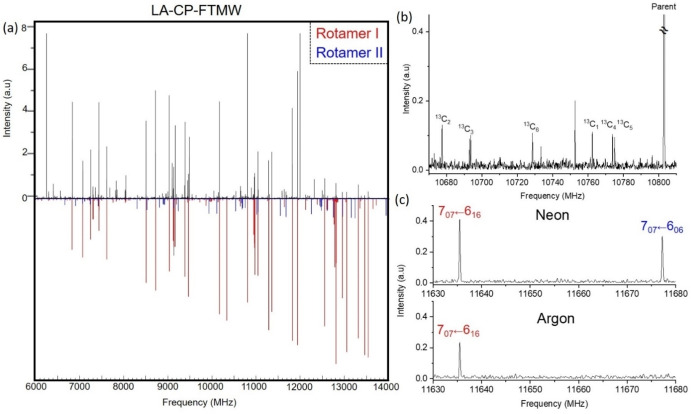
(a) The broadband LA‐CP‐FTMW rotational spectrum of piracetam in the 6000–14000 MHz range, together with the simulated rotational spectra for the two conformers detected. (b) Zoomed‐in section (10670–10810 MHz) of the broadband LA‐CP‐FTMW spectrum showing the isotopologues in comparison to the parent species of *Exo‐I*. (c) Zoomed‐in section (10630–10680 MHz) of the broadband LA‐CP‐FTMW spectrum showing the difference in intensity of selected rotational transitions of *Exo‐I* and *Endo‐I* upon changing the carrier gas from neon (top) to argon (bottom).

We utilized our LA‐CP‐FTMW spectrometer[^23^, ^24^, ^25^, ^26^, ^27^, ^28^] to capture the microwave spectrum of piracetam within the 6.0–14.0 GHz frequency range, as depicted in Figure 2a. The spectrum reveals highly intense rotational lines. Initially, lines associated with known photofragment species and water clusters were identified and excluded.[^27^, ^29^, ^30^] The remaining spectrum still displayed numerous rotational transitions broader than usual, probably due to the hyperfine structure of piracetam. For the analysis, JB95^[31]^ and *Assignment and Analysis of Broadband Spectra* (AABBS)[^32^, ^33^, ^34^, ^35^] software packages were used. Upon initial inspection, the distinct pattern of an *a*‐type *R*‐branch progression from a predominant rotameric species was swiftly recognized. The initial set of rotational constants derived from a rigid rotor analysis^[35]^ facilitated the rapid identification of *b*‐type transitions with new predictions. In a second step, all rotational transitions were submitted to the analysis employing Watson's asymmetric top semirigid‐rotor Hamiltonian in *A*‐reduction and I^r^‐representation. A total of 61 rotational transitions was assigned and measured (see Table S05), enabling the determination of very precise rotational constants (*A*=2340.4991, *B*=1006.68196 and *C*=808.98760 MHz) for this first rotamer, labelled as rotamer I.

After removing this rotamer‘s rotational lines, weak transitions remained unassigned in the spectrum, so we looked for other species. Again, a distinct pattern of a weak *a*‐type *R*‐branch progression was observed. No *b*‐ or *c*‐type transitions were in the rotational spectrum. 19 rotational transitions were assigned and measured (see Table S06 in the Electronic Supplementary Information, ESI). The rotational constants derived from a rigid rotor analysis for this rotamer II are collected in Table 1.

### Conformational Assignment

After removing the rotational lines due to the two detected rotamers, several weak lines remained in the spectrum, but they could not be attributed to any additional conformer of piracetam. They belong to the isotopic species of piracetam, which will be discussed later. Thus, two stable conformers are present in the supersonic expansion.

The comparison between the experimental and theoretical rotational constants in Table 1 shows a perfect match between theory and experiment for the most stable structure, *Exo‐I*, with rotamer I. Additionally, for rotamer I *a*‐type rotational transitions are dominant in the spectrum, *b*‐type transitions are considerably weaker, *c*‐transitions are even weaker, and only a few transitions of the latter were observed. It agrees with the selection rules shown in Table 1 for *Exo‐I*, which shows a large value of *μ_a_*, a considerably lower value of *μ*_*b*,_ and an even lower value of *μ_c_*, particularly considering that, in this technique, the intensity is proportional to the square of the dipole moment.^[36]^ Note that the observation of *c*‐type transitions is another point for validating this conformer, as for *Endo‐I* such transitions would not be observed due to a negligible dipole moment. For rotamer II, Table 1 shows again a perfect match between theory and experiment for *Endo‐I* structure. The non‐observation of *b*‐type transitions is attributed to the low dipole moment and a low abundance of this conformer (*vide infra*). Therefore, rotamers I and II are assigned to *Exo‐I* and *Endo‐I* structures, respectively.

### Hyperfine Structure

The diagonal elements of the nuclear quadrupole coupling tensor for each ^14^N can be used as a more precise assignment, as they largely depend on the chemical environment of each nitrogen atom. The two ^14^N nuclei in the amine groups of piracetam have a non‐zero quadrupole moment (*I*=1), which interacts with the electric field gradient at these nuclei sites, resulting in a hyperfine structure for all rotational transitions.[^37^, ^38^, ^39^] The analysis of the nuclear quadrupole coupling constants (*χ_aa_*, *χ_bb_*, *χ_cc_*) provides information about the electronic environment of the nitrogen nuclei, serving as their fingerprint, which assures us of the structure beyond doubt. A total of 121 components were finally measured for rotamer I using a Watson‘s *A*‐reduced rigid rotor Hamiltonian^[40]^ supplemented with a term to account for the nuclear quadrupole coupling interaction,[^41^, ^42^] and the obtained rotational parameters are listed in the first column of Table 1. Regarding rotamer II, its low intensity did not allow us to perform a proper fit.

Once the nuclear quadrupole constants were experimentally determined, they were compared with those predicted for the most stable conformer. As can be seen in Table 1, there is excellent agreement between the experimental and theoretical values of the nuclear quadrupole coupling constants for *Exo‐I*, confirming the previous assignment.

Interestingly, the calculated rotational constants using B3LYP‐GD3 agree better with those obtained experimentally, but the opposite is true for the quadrupole constants, for which MP2 seem to perform extremelly well.

### Isotopic Species

As previously stated, after removing the lines due to the two detected rotamers, several weak lines remained in the spectrum, which were not due to other conformers of piracetam. Since the most stable conformer *Exo‐I* is abundant, the observed transitions are very intense, and the remaining lines could be due to transitions of the ^13^C isotopologs in their natural abundance (1.109 %). Additionally, the weak transitions were on the low‐frequency side of each transition of the detected *Exo‐I* conformer. Thus, several rotational transitions were fitted for the six carbon isotopic species. Unfortunately, the lower natural abundance of the isotopic species of nitrogen and oxygen precluded us from making any reliable fit for these two isotopologues. Figure 2b shows some of the transitions belonging to all the carbon isotopic species, Table 2 shows their rotational constants, and Tables S07 to S12 collect the measured frequencies.

**Table 2:** Experimental spectroscopic parameters for the observed isotopologues of *Exo‐I* conformer.

Parameters	Parent	^13^C_1_	^13^C_2_	^13^C_3_	^13^C_4_	^13^C_5_	^13^C_6_
*A* ^[a]^	2340.53783(173)^[e]^	2310.33(50)	2325.615(99)	2335.487(143)	2333.015(107)	2327.592(155)	2340.32(34)
*B*	1006.68679(112)	1004.7801(79)	994.64318(266)	995.0014(34)	1004.23736(228)	1002.8699(42)	998.8908(96)
*C*	809.00161(107)	804.7740(55)	799.73764(222)	801.64310(305)	806.62406(243)	807.8521(33)	803.9184(64)
*ΔJ* ^[b]^	0.1580(119)	–	–	–	–	–	–
*N* ^[c]^	121	7	8	11	9	11	10
*σ* ^[d]^	33.2	37.8	20.3	33.6	26.7	37.2	54.4

[a] *A*, *B*, and *C* represent the rotational constants (in MHz). [b] ΔJ is the quartic centrifugal distortion constants (in MHz). [c] N is the number of measured transitions. [d] σ is the rms deviation of the fit (in kHz). [e] Standard error in parentheses in units of the last digit.

**Figure 3:**
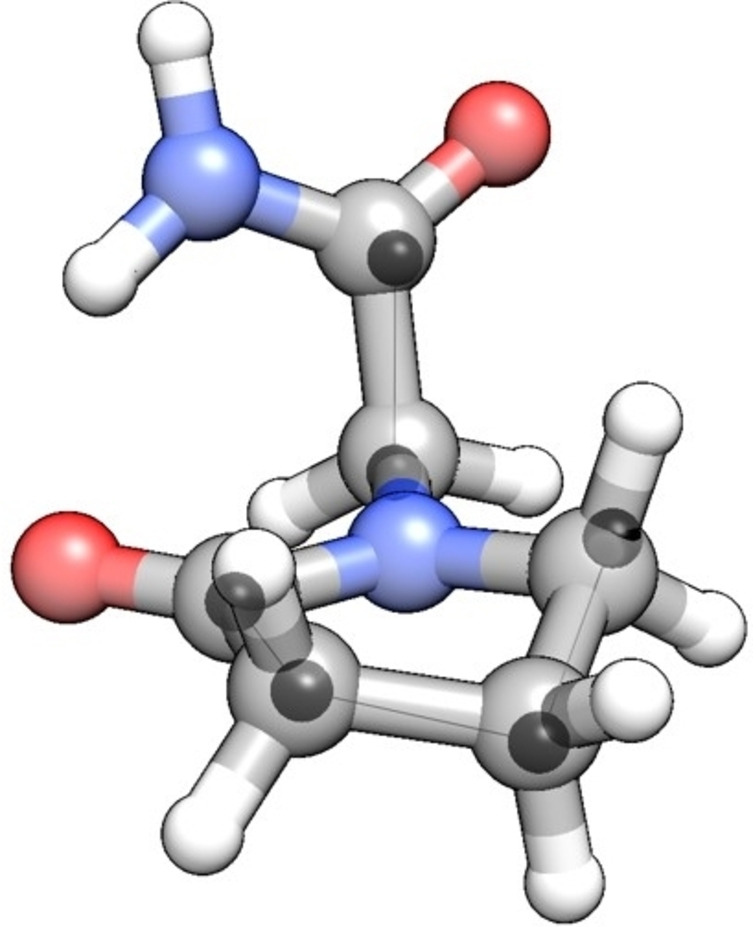
The calculated structure of *Exo‐I* superimposed with the determined r_s_ positions of the carbon atoms (dark spheres).

From the set of observed rotational constants in Table 2, the substitution structure (r_s_) of the heavy atom skeleton was derived. Using the Kraitchman equations,^[43]^ the cartesian coordinates of the substituted atoms are determined in the principal axis system. The r_s_ substitution coordinates are given in Table S13. Figure 3 compares the calculated structure and the substitution structure for the carbon atoms.

### Conformational Partial Interconversion

Assuming a Boltzmann distribution at ~300 K before the supersonic expansion and that the population “freezes” immediately during the expansion, the population ratio between the most stable conformer and the second most stable should be ~1 : 0.6, according to the calculations. It drastically deviates from that derived experimentally from the observed intensities, as shown in Figure 2a. In fact, the intensity of the rotational transitions belonging to *Endo‐I* is only about 2 % of that of *Exo‐I*, which would imply a very low population for *Endo‐I*. This discrepancy cannot be due to bad energy estimations of the calculations, as different methodologies set *Endo‐I* energetically close to *Exo‐I*. Moreover, the structures are very similar, and the calculated relative energy difference should be reliable.

**Figure 4:**
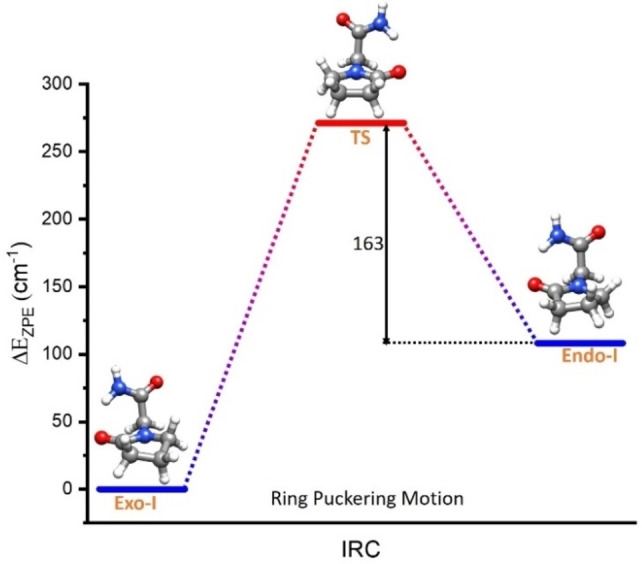
Comparison of the relative energies of *Exo‐I* and *Endo‐I* structures of piracetam and the transition state (TS) that connects both ring configurations calculated at B3LYP‐GD3BJ/6‐311G++(d,p) and corrected with harmonic zero point energy. The energy barrier is small enough to lose a considerable population of *Endo‐I* conformer during supersonic expansion due to the collisions with the carrier gas.

To explain this “loss” of intensity (and, therefore, abundance) of *Endo‐I*, we considered a possible conformational relaxation of this conformer to the more stable *Exo‐I*. Conformational relaxation occurs through collisions with the noble carrier gas in the adiabatic expansion when the interconversion barrier is low enough. By examining the behavior of a variety of systems, Ruoff et al.[^44^, ^45^] noted that efficient relaxation occurs for conformational species separated by an energy barrier lower than 400 cm^−1^. Similar behavior has been observed for proteinogenic amino acids.^[46]^ Because the structures of *Exo‐I* and *Endo‐I* are separated by ring puckering, we performed a QST3 calculation^[47]^ to obtain the transition structure connecting both conformers. Figure 4 shows the results obtained. As can be seen, the data indicate that the interconversion, which occurs when transitioning from the *exo* to the *endo* configuration of the ring with the transition state being a planar ring, crosses a considerably low barrier (less than 200 cm^−1^ using B3LYP‐D3/6‐311++G(d,p) and ~350 cm at MP2/6‐311++G(d,p)). It is the reason for the partial conformational loss, explaining why *Endo‐I* structure‘s population is lower than expected.

To confirm this population loss of *Endo‐I* structure through interconversion, we performed an additional experiment using Ar instead of Ne as a buffer gas. We can increase the interconverted population by using Ar as the carrier gas because collisions with Ar are more energetic than with Ne. As relaxation is caused by binary collisions with the inert gas atoms, heavier inert gas atoms result in more effective collisions. Therefore, the more energetic collisions with Ar move part of the population from *Endo‐I* conformer to *Exo‐I*, resulting in the complete depopulation of E*ndo‐I* conformer, as seen in Figure 1c.

With all the data above, we have both experimental and theoretical evidence supporting the existence of conformational relaxation. Another important point is that these results validate that *Exo‐I* conformer is more stable than *Endo‐I* conformer as the calculations predict. Otherwise, if *Exo‐I* were less stable, this structure would be depleted. It is further proof of the importance of experiments for validating computational calculations. Additionally, this is the third proof confirming the previous assignment of rotamer I corresponding to *Exo‐I* and rotamer II corresponding to the *Endo‐I*.

### Intramolecular Interactions

**Figure 5:**
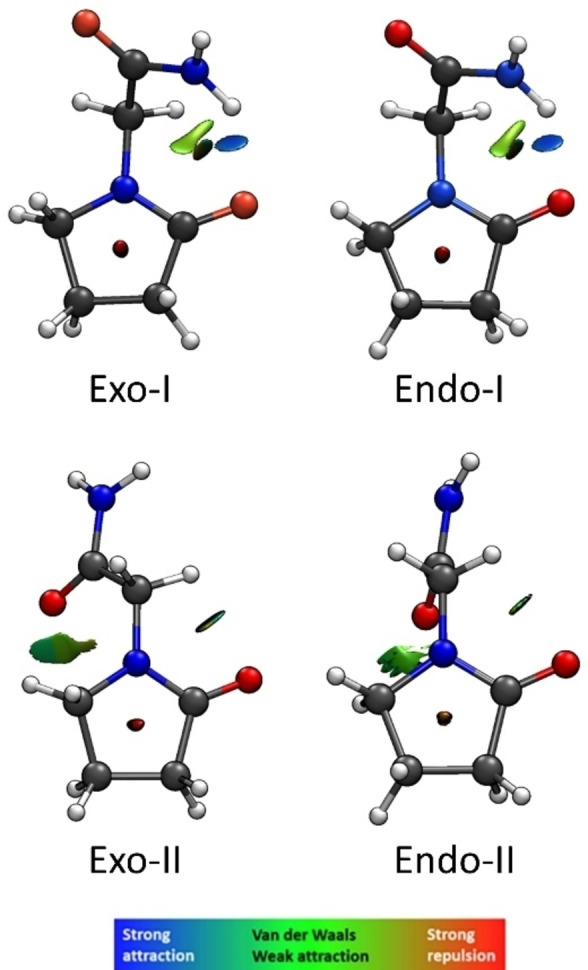
NCIPlot results of the four most stable conformers of piracetam. Grey corresponds to carbons, blue to nitrogen, red to oxygen and white to hydrogen. Red surfaces correspond to repulsive forces, blue surfaces to moderate attractive forces, and green surfaces to weak attractive interactions. A contour value of 0.35 was used for the representation.

Once the conformational assignment process is completed, we evaluate the intramolecular interactions governing the detected structures. Figure 5 shows an NCI (Non‐Covalent Interactions) calculation performed with the NCIPlot software[^48^, ^49^] for the four calculated structures. The results show that *Exo‐I* and *Endo‐I* conformers are primarily stabilized by a relatively strong N−H⋅⋅⋅O=C hydrogen bond between the amide group and the ring carbonyl oxygen (blue color in Figure 5). Additionally, there is a slightly weaker interaction between the methyl group near the amide and the carbonyl in the pyrrolidine ring (green color in Figure 5). There is also an C−H⋅⋅⋅O=C interaction between the same methyl group and the amide carbonyl, but it is weak and can only be seen by increasing the contour cut off value.

For Exo‐II and Endo‐II conformers, the structures are stabilized by a C−H⋅⋅⋅O=C bond between the methyl group of the ring at the C_γ_/C_1_ position and the amide carbonyl (green color in Figure 5). There is also another moderate C−H⋅⋅⋅O=C interaction between the methyl group adjacent to the amide and the carbonyl in the pyrrolidine ring (green color in Figure 5).

### Conformers of Piracetam

In this section, we compare our results with those reported so far. Gas phase results showed that an intramolecular hydrogen bond between the amine group and the carbonyl of the pyridine ring stabilizes one stable conformer of piracetam.^[14]^ Our results show that there are two stable conformers of piracetam, and that both agree with the intramolecular interactions that stabilize the conformers. We also note that, besides the hydrogen bond interaction between the NH_2_ and the carbonyl, the structure is also stabilized by a weak intramolecular interaction between the ring‘s CH_2_ and the carbonyl of the pyrrolidine ring. Additionally, the geometry reported so far shows that the detected conformer contains the ring in an *endo* disposition.^[14]^ However, our results indicate two conformers, one in the *endo* disposition as reported, but the most stable conformer being in an *exo* disposition. These results prove microwave spectroscopy‘s power to discern even between similar structures.

### Biological Implication

**Figure 6:**
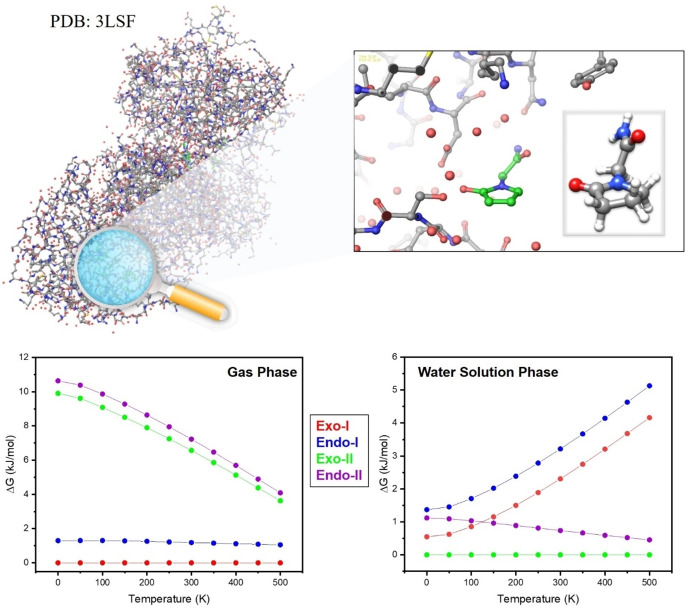
Top: a comparison between the calculated structure of piracetam (*Endo‐II/Exo‐II*) in isolated conditions (grey frame) and its structure within the crystalline structure of the S1S2 domain of the AMPA GluA2 receptor (PDB: 3LSF)^[50]^. Bottom: variation of the Gibbs free energy with temperature relative to the global minimum in vacuum conditions and in water as implicit solvent using the IEFPCM model at B3LYP‐GD3BJ/6‐311++G(d,p).

Figure 6 shows a crystalline structure of the S1S2 domain of the AMPA GluA2 receptor with piracetam (PDB: 3LSF),[^50^, ^51^] and a close‐up view of one of the interaction regions. The structure adopted by piracetam when interacting with the nearby amino acids within the domain resembles *Exo‐II* and *Endo‐II* structures, more precisely in their transition state. It seems logical that the crystal structure does not show the 3D‐disposition of *Exo‐I* and *Endo‐I* within the pocket, as such structures would not interact with the ligand because the amino group not being accessible to interact with the amino acids within the pocket. Thus, the structure adopts a disposition closer to that of *Exo‐II* and *Endo‐II* conformers, where the amino group is free to interact with the surrounding amino acids. Additionally, we need to consider the Gibbs free energy at room temperature. Curiously, Figure 6 shows significant stabilization of *Exo‐II* and *Endo‐II* structures with increasing temperature, and there is a noteworthy population of *Endo‐II* and *Exo‐II* conformers at body temperature. Furthermore, when the calculations use water as a solvent, *Exo‐II* and *Endo‐II* structures are dominant. Therefore, it would not be surprising to observe these structures in the crystal protein since those are the most stable structures and have the functional groups “free” to interact with the amino acids within the receptor site. Furthermore, because at room temperature, the internal energy of the molecules is larger than the barrier separating the *endo*/*exo* ring configurations, the molecule would be continuously switching between both forms, so it is not surprising to see the ring planar.

## Conclusions

The precise structural determination of piracetam (2‐oxo‐1‐pyrrolidineacetamide), a nootropic drug belonging to the racetam family, represents a crucial milestone in pharmaceutical research and drug development. Thus, in this work, we used rotational spectroscopy to obtain an accurate picture of piracetam. The combination of computational chemistry and rotational spectroscopy has allowed the characterization of the most relevant conformers of the piracetam monomer in the gas phase. It led to the unequivocal identification of the intrinsic structure of two conformers, which are two of the four structures predicted as most stable by quantum mechanical calculations, obtaining their rotational constants.

The NCI analysis allowed to evaluate the intramolecular interactions of the two most stable conformers characterized experimentally. The results show that the two observed conformers are stabilized by an N−H⋅⋅⋅O=C hydrogen bond between the amide group and the carbonyl oxygen of the ring, as well as an intramolecular C−H⋅⋅⋅O=C interaction between the methyl group near the acetamide group and the carbonyl of the pyrrolidone nucleus. To a lesser extent, there is an intramolecular C−H⋅⋅⋅O=C interaction between the same methyl group and the carbonyl of the acetamide group. The two conformers differ in the *exo* and *endo* disposition of the ring.

Conformational relaxation from the *endo* to the *exo* disposition occurring during the supersonic expansion process is also demonstrated, confirming that the most stable structure of piracetam in isolated conditions has the pyrrolidone ring in an *exo* configuration. The Kraitchman equations for the most stable species determine the r_s_ positions of the carbon atoms.

Finally, the most stable conformers of piracetam in isolated conditions are compared with those calculated using water as a solvent and with its structure within the crystalline structure of the S1S2 domain of the AMPA GluA2 receptor.

## Materials and Methods

### Experimental Section

A commercial sample of piracetam (Cymit, 98 %), a crystalline white solid with a melting point of 425 K, was used without any further purification. It was finely pulverized and intimately mixed with a small amount of commercial binder (Peoval). The resulting fine powder was subjected to hydraulic pressing to form a solid rod. Posteriorly, the sample was placed in the ablation nozzle. The spectrum was recorded using a broadband chirped‐pulse Fourier‐transform microwave (CP‐FTMW) spectrometer in the frequency range of 6–14 GHz.[^52^, ^53^] To generate particles of piracetam in gas phase, we employed a picosecond Nd:YAG laser (20 ps pulse width, 355 nm) with a fluence of 16 mJ. The vaporized products were diluted at 10 bar of stagnation pressure in different carrier gases (Ne and Ar). After its vaporization, we used a pulsed valve connected to a vacuum chamber of the spectrometer with a repetition rate of 2 Hz to produce a supersonic expansion and cool the molecular system. A 24 GS s^−1^ arbitrary waveform generator (AWG) creates a fast chirp microwave pulse of 4 μs, which is subsequently amplified using a 300 W traveling wave tube amplifier to macroscopically polarize the molecules at the spectral work region. Up to 80 k individual free induction decay (FID) of the macroscopic polarization was recorded in the time domain using a 50 GS s^−1^ oscilloscope. 4 FIDs were acquired on each valve cycle. The overall FIDs were averaged in the time domain, followed by a Fast Fourier transformation to transform it into the frequency domain.

### Computational Methods

The conformational landscape of piracetam was explored using a systematic procedure. In the first step, we evaluated all possible structures that are minima on the potential energy hypersurface through fast molecular mechanics methods using “Large scales Low Mode” and Monte Carlo‐based search algorithms.^[54]^ Subsequently, every molecular structure was geometrically re‐optimized using the DFT level of theory at B3LYP‐GD3BJ[^20^, ^55^, ^56^, ^57^] with the 6‐311++G(d,p) basis set.[^21^, ^58^] All the optimized structures were confirmed as local minima in the potential energy Surface (PES) by checking that their Hessian matrix had no imaginary eigenvalues. We considered the harmonic approximation in frequency calculation to obtain the zero‐point energy correction (ZPE). In order to find the structures of the transition states we used the Synchronous Transit‐guided Quasi‐Newton method^[59]^ using QST3. To emulate aqueous conditions, we reoptimized the previously obtained gas‐phase structures using the implicit solvation model IEFPCM with water as the solvent, employing the B3LYP‐GD3BJ/6‐311G++(d,p) level of theory. All the quantum chemical computations were performed in Gaussian16 package.^[60]^ The results are summarized in Tables S1 to S14.

## Supporting Information

Broadband rotational spectrum of piracetam using Ne and Ar. Cartesian coordinates in Angstroms (Å) of the conformers of piracetam; experimental transition frequencies (v/MHz) together with the corresponding observed‐calculated differences (Δv/kHz) for parent species and isotopoloques; Cartesian coordinates in Angstroms (Å) for the r_s_ structure of Exo‐I conformer of piracetam, derived from the ^13^C isotopologues substitution.

## Conflict of Interests

The authors declare no conflict of interest.

## Supporting information

As a service to our authors and readers, this journal provides supporting information supplied by the authors. Such materials are peer reviewed and may be re‐organized for online delivery, but are not copy‐edited or typeset. Technical support issues arising from supporting information (other than missing files) should be addressed to the authors.

Supporting Information

## Data Availability

The data that support the findings of this study are available in the supplementary material of this article.
