# Supplementary material for: Elucidating the Three‐Dimensional Structure of Piracetam through Rotational Spectroscopy
Source: ChemistryOpen. 2025 Mar 15;14(8):e202400490. doi: 10.1002/open.202400490 (PMC12368879; doi:10.1002/open.202400490)
Supplement: Supplementary file 1 — Supporting Information [file OPEN-14-e202400490-s001.pdf]

ChemistryOpen

Supporting Information

Elucidating the Three-Dimensional Structure of Piracetam through Rotational Spectroscopy

S. Mato, S. Municio, J. L. Alonso, E. R. Alonso, and I. León*

Supporting Information

Elucidating the Three-Dimensional Structure of Piracetam through Rotational Spectroscopy

*S.Mato, S.Municio, J.L. Alonso, E.R. Alonso, I.León**

Figure S01. The broadband LA-CP-FTMW rotational spectrum of piracetam in the 6000-14000 MHz range, together with the simulated rotational spectra for the two conformers detected using Ne (top) and Ar (bottom).

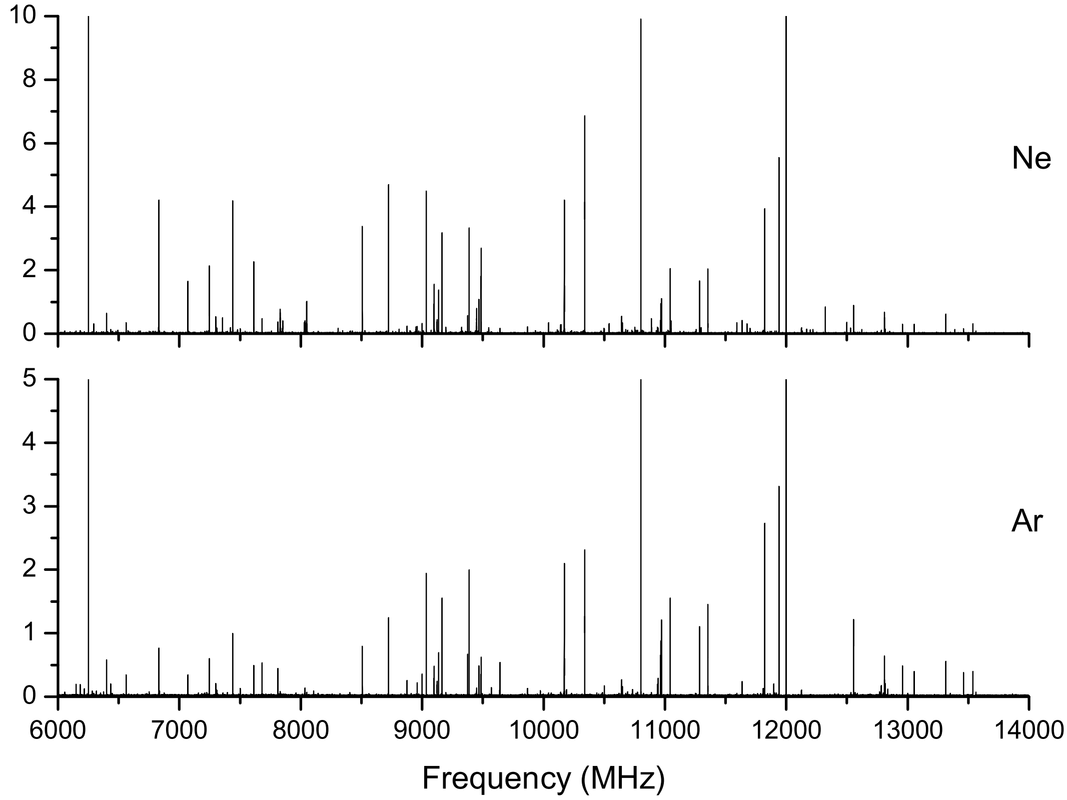

Table S01. Cartesian coordinates in Angstroms (Å) of *Exo-I* conformation at B3LYP-GD3BJ/6-311++G(d,p).

atom	x	y	z
C	-1.044028	-1.479261	-0.073522
C	-2.484584	-0.981335	-0.298065
C	-2.324084	0.534395	-0.494292
H	-0.559984	-1.780599	-1.010496
H	-0.985599	-2.319663	0.621144
H	-2.962697	-1.479158	-1.141467
H	-3.081836	-1.181443	0.594121
H	-2.220422	0.808358	-1.549349
H	-3.134827	1.133439	-0.080072
C	-1.008548	0.868017	0.192062
O	-0.548606	1.983421	0.405985
N	-0.381241	-0.304206	0.490508
C	0.968093	-0.345356	1.026598
H	1.130925	-1.303182	1.517477
H	1.063394	0.459936	1.758585
H	2.70859	1.209411	-1.394853
N	2.051274	1.036177	-0.650435
H	1.378703	1.745519	-0.382063
C	2.04478	-0.18669	-0.060251
O	2.806574	-1.0928	-0.352074

Table S02. Cartesian coordinates in Angstroms (Å) of *Endo-I* conformation at B3LYP-GD3BJ/6-311++G(d,p).

atom	x	y	z
C	1.157805	-1.499141	0.159639
C	2.34699	-0.955742	-0.658389
C	2.43099	0.527417	-0.262891
H	0.567641	-2.233682	-0.391868
H	1.479689	-1.958702	1.102519
H	2.124269	-1.042729	-1.723948
H	3.264817	-1.50942	-0.461775
H	3.103294	0.694729	0.585096
H	2.745402	1.19069	-1.067854
C	1.019604	0.867359	0.190082
O	0.531479	1.982733	0.33403
N	0.365625	-0.300782	0.428296
C	-0.970782	-0.343674	0.998992
H	-1.12123	-1.303856	1.488637
H	-1.058548	0.458795	1.736695
H	-1.38814	1.734718	-0.413436
N	-2.089772	1.040703	-0.647941
H	-2.74645	1.208108	-1.394181
C	-2.066813	-0.185323	-0.067293
O	-2.832539	-1.093166	-0.344431

Table S03. Cartesian coordinates in Angstroms (Å) of *Exo-II* conformation at B3LYP-GD3BJ/6-311++G(d,p).

atom	x	y	z
C	1.032304	-1.377658	0.367949
C	2.491047	-0.992907	0.055413
C	2.375369	0.404647	-0.571424
H	0.557342	-1.905372	-0.464246
H	0.941643	-1.990561	1.269024
H	2.972404	-1.721454	-0.596979
H	3.06483	-0.939411	0.9837
H	3.206175	1.073409	-0.347926
H	2.268322	0.358875	-1.659885
C	1.071142	0.961983	-0.018301
O	0.666143	2.108536	-0.08561
N	0.395088	-0.07772	0.569732
C	-0.978632	0.07376	0.955771
H	-1.207847	-0.590115	1.795573
H	-1.12097	1.10576	1.286334
H	-3.492342	0.693634	0.844835
N	-3.25406	0.085748	0.077895
H	-3.925062	-0.013991	-0.668824
C	-1.953594	-0.231638	-0.193616
O	-1.600831	-0.753048	-1.233108

Table S04. Cartesian coordinates in Angstroms (Å) of *Endo-II* conformation at B3LYP-GD3BJ/6-311++G(d,p).

atom	x	y	z
C	0.926677	-1.28118	0.659625
C	2.213005	-1.266103	-0.188756
C	2.57486	0.222647	-0.295211
H	0.226995	-2.053937	0.343514
H	1.141752	-1.41146	1.729449
H	1.990541	-1.665453	-1.180269
H	3.002327	-1.874718	0.252801
H	3.247813	0.546851	0.505667
H	3.029423	0.509361	-1.242875
C	1.247631	0.946872	-0.106315
O	1.002446	2.117325	-0.342269
N	0.362512	0.043312	0.417286
C	-0.975685	0.414737	0.772653
H	-1.199617	0.116797	1.80488
H	-1.034583	1.504993	0.714487
H	-4.014642	-0.04123	-0.594044
N	-3.287308	0.289216	0.021647
H	-3.44483	1.148552	0.522549
C	-2.030083	-0.216902	-0.147155
O	-1.778702	-1.12081	-0.919948

Table S05. Measured rotational transitions for rotamer I.

J´	K´a	K´c	I´	F´	J´´	K´´a	K´´c	I´´	F´´	ν_{obs}	ν_{obs}-ν_{cal}
3	1	3	4	5	2	0	2	3	4	6294.687	-0.021
3	1	3	4	4	2	0	2	3	3	6294.962	0.025
3	1	3	3	4	2	0	2	2	3	6295.297	-0.033
6	2	5	6	7	6	0	6	6	7	6780.744	0.072
6	2	5	7	6	6	0	6	7	6	6781.514	0.061
4	1	4	3	4	3	1	3	3	4	6828.861	0.000
4	1	4	5	6	3	1	3	4	5	6830.231	0.028
4	1	4	4	5	3	1	3	4	5	6831.336	-0.015
4	3	1	5	6	4	2	2	5	6	6945.208	-0.051
4	3	1	4	4	4	2	2	4	4	6945.513	-0.049
4	0	4	3	4	3	0	3	3	4	7069.818	-0.012
4	0	4	5	6	3	0	3	4	5	7070.617	-0.018
4	0	4	4	5	3	0	3	3	4	7070.878	0.052
4	0	4	4	4	3	0	3	4	4	7071.417	-0.066
7	2	6	8	9	7	1	7	8	9	7210.398	-0.030
4	2	3	5	6	3	2	2	4	5	7246.858	0.060
4	3	2	5	5	3	3	1	4	4	7299.565	-0.003
4	3	2	5	6	3	3	1	4	5	7299.799	-0.007
4	3	2	4	5	3	3	1	3	4	7300.068	-0.006
4	3	1	5	5	3	3	0	4	4	7307.427	-0.021
4	3	1	5	6	3	3	0	4	5	7307.700	0.011
4	3	1	4	5	3	3	0	3	4	7307.911	-0.032
4	2	2	5	6	3	2	1	4	5	7438.749	-0.054
6	3	4	6	7	6	2	5	6	7	7500.786	-0.029
6	3	4	7	8	6	2	5	7	8	7501.172	-0.008
4	1	3	5	5	3	1	2	4	5	7612.630	-0.002
4	1	3	5	6	3	1	2	4	5	7613.177	0.074
4	1	3	4	3	3	1	2	3	3	7613.696	-0.048
4	1	4	5	6	3	0	3	4	5	7758.136	0.079
2	2	1	3	2	1	1	0	0	1	7829.254	0.036
2	2	1	1	2	1	1	0	0	1	7829.526	0.028
2	2	1	3	4	1	1	0	2	3	7830.454	-0.026
2	2	1	3	3	1	1	0	2	2	7830.697	-0.073
2	2	1	2	3	1	1	0	1	2	7830.997	-0.007
2	2	1	3	2	1	1	0	1	1	7831.304	0.021
2	2	1	1	2	1	1	0	1	2	7831.595	0.001
2	2	0	1	2	1	1	0	0	1	7849.963	0.016
2	2	0	3	4	1	1	0	2	3	7850.862	-0.031
2	2	0	3	3	1	1	0	2	2	7851.203	0.050
2	2	0	1	2	1	1	0	1	2	7852.064	0.022
2	2	1	2	3	1	1	1	1	2	8027.510	0.020
2	2	1	3	4	1	1	1	2	3	8028.448	-0.004
2	2	1	3	2	1	1	1	0	1	8029.256	0.019
2	2	1	1	2	1	1	1	0	1	8029.550	0.033
5	0	5	5	5	4	1	4	4	4	8035.309	0.010

5	0	5	6	7	4	1	4	5	6	8035.569	0.012
2	2	0	2	3	1	1	1	1	2	8047.823	0.017
2	2	0	3	4	1	1	1	2	3	8048.841	-0.025
2	2	0	1	2	1	1	1	0	1	8049.947	-0.019
5	1	5	4	5	4	1	4	4	5	8506.266	0.008
5	1	5	4	5	4	1	4	3	4	8507.683	-0.019
5	1	5	5	6	4	1	4	5	6	8508.893	0.002
5	0	5	4	5	4	0	4	4	5	8721.938	-0.054
5	0	5	6	7	4	0	4	5	6	8722.951	-0.029
5	0	5	5	6	4	0	4	5	6	8723.921	-0.035
5	2	4	6	7	4	2	3	5	6	9033.106	0.022
5	4	2	6	7	4	4	1	5	6	9123.668	0.052
5	4	1	6	7	4	4	0	5	6	9124.241	0.058
5	3	3	6	7	4	3	2	5	6	9135.582	-0.035
5	3	3	5	6	4	3	2	4	5	9135.752	0.009
5	3	2	6	7	4	3	1	5	6	9162.812	0.020
5	1	5	6	7	4	0	4	5	6	9195.091	0.011
5	1	5	5	6	4	0	4	4	5	9195.520	-0.008
5	1	5	5	5	4	0	4	4	4	9195.718	0.063
5	2	3	6	7	4	2	2	5	6	9387.869	-0.030
3	2	2	2	3	2	1	1	1	2	9448.101	-0.022
3	2	2	4	5	2	1	1	3	4	9448.396	-0.009
3	2	2	4	4	2	1	1	3	3	9448.668	0.001
3	2	2	3	4	2	1	1	2	3	9448.996	-0.028
3	2	2	4	3	2	1	1	2	2	9449.213	0.011
5	1	4	6	6	4	1	3	5	6	9468.510	0.004
5	1	4	6	7	4	1	3	5	6	9468.899	-0.037
3	2	1	4	5	2	1	1	3	4	9548.961	-0.049
3	2	1	4	4	2	1	1	3	3	9549.212	0.009
3	2	1	3	4	2	1	1	2	3	9549.430	0.051
6	0	6	7	8	5	1	5	6	7	9866.985	-0.063
3	2	2	3	4	2	1	2	2	3	10041.002	0.033
3	2	2	4	5	2	1	2	3	4	10041.837	-0.036
3	2	2	2	3	2	1	2	1	2	10042.389	0.030
3	2	1	3	4	2	1	2	2	3	10141.261	-0.063
3	2	1	2	3	2	1	2	1	2	10143.034	-0.011
6	1	6	5	6	5	1	5	5	6	10169.351	0.009
6	1	6	7	8	5	1	5	6	7	10170.756	-0.039
6	1	6	6	7	5	1	5	6	7	10172.116	0.012
6	0	6	5	6	5	0	5	5	6	10337.943	-0.034
6	0	6	7	8	5	0	5	6	7	10339.114	-0.034
6	0	6	6	7	5	0	5	5	6	10339.309	-0.008
6	0	6	6	7	5	0	5	6	7	10340.275	-0.019
6	1	6	7	8	5	0	5	6	7	10642.935	0.039
6	1	6	6	7	5	0	5	5	6	10643.259	0.031
6	2	5	7	8	5	2	4	6	7	10802.920	0.003
6	4	3	7	8	5	4	2	6	7	10962.497	-0.020
6	4	3	6	7	5	4	2	5	6	10962.677	0.025

6	4	2	7	8	5	4	1	6	7	10965.107	0.063
6	3	4	7	8	5	3	3	6	7	10972.931	0.028
6	3	3	7	8	5	3	2	6	7	11043.344	0.002
4	2	2	5	5	3	1	2	4	4	11257.821	0.009
6	1	5	7	8	5	1	4	6	7	11286.417	-0.037
3	2	1	3	4	2	0	2	2	3	11298.196	0.010
3	2	1	4	5	2	0	2	3	4	11298.866	0.005
3	2	1	2	3	2	0	2	1	2	11299.258	0.020
6	2	4	7	8	5	2	3	6	7	11355.238	0.005
7	0	7	8	9	6	1	6	7	8	11635.480	-0.039
7	1	7	6	7	6	1	6	6	7	11819.639	0.016
7	1	7	8	9	6	1	6	7	8	11821.112	-0.018
7	1	7	7	8	6	1	6	7	8	11822.554	0.049
7	0	7	6	7	6	0	6	6	7	11937.928	-0.008
7	0	7	8	8	6	0	6	7	7	11939.311	0.010
7	0	7	7	8	6	0	6	7	8	11940.558	0.011
7	1	7	8	9	6	0	6	7	8	12124.881	0.005
7	1	7	7	8	6	0	6	6	7	12125.147	0.040
7	2	6	7	8	6	2	5	6	7	12553.961	0.007
7	3	5	8	9	6	3	4	7	8	12807.549	-0.051
7	4	4	8	9	6	4	3	7	8	12807.988	0.028
7	4	3	8	9	6	4	2	7	8	12816.275	0.006
7	3	4	8	9	6	3	3	7	8	12958.529	-0.034
7	1	6	8	9	6	1	5	7	8	13053.733	-0.058
7	2	5	8	8	6	2	4	7	7	13315.771	0.030
8	0	8	9	10	7	1	7	8	9	13351.912	-0.017
8	1	8	9	10	7	1	7	8	9	13461.021	0.002
8	0	8	9	9	7	0	7	8	8	13537.593	0.027

Table S06. Measured rotational transitions for rotamer II.

J´	K´a	K´c	J´´	K´´a	K´´c	ν_{obs}	ν_{obs}-ν_{cal}
4	1	4	3	1	3	6668.045	0.024
4	2	2	3	2	1	7239.628	0.002
4	1	3	3	1	2	7420.449	0.090
5	1	5	4	1	4	8307.736	0.068
5	0	5	4	0	4	8528.236	0.048
5	2	4	4	2	3	8810.603	0.009
5	2	3	4	2	2	9133.078	0.027
5	1	4	4	1	3	9233.607	0.002
6	1	6	5	1	5	9933.981	0.007
6	0	6	5	0	5	10111.903	-0.016
6	2	5	5	2	4	10539.795	0.022
6	3	3	5	3	2	10752.682	-0.055
6	1	5	5	1	4	11013.267	0.022
6	2	4	5	2	3	11047.168	-0.023
7	1	7	6	1	6	11548.028	-0.061
7	0	7	6	0	6	11677.229	0.029
7	2	6	6	2	5	12251.893	-0.015
7	1	6	6	1	5	12748.508	-0.066
8	0	8	7	0	7	13238.408	-0.024

Table S07. Measured rotational transitions for the $^{13}C_1$ isotopologue of *Exo-I* conformer of piracetam.

J´	K´a	K´c	J´´	K´´a	K´´c	ν_{obs}	ν_{obs}-ν_{cal}
4	1	4	3	1	3	6799.576	-0.039
5	1	5	4	1	4	8468.340	-0.034
5	2	4	4	2	3	9000.519	-0.043
5	3	2	4	3	1	9136.324	0.025
6	1	6	5	1	5	10122.484	-0.023
6	2	5	5	2	4	10762.406	0.020
7	1	7	6	1	6	11763.757	0.062

Table S08. Measured rotational transitions for the $^{13}C_2$ isotopologue of *Exo-I* conformer of piracetam.

J´	K´a	K´c	J´´	K´´a	K´´c	ν_{obs}	ν_{obs}-ν_{cal}
5	1	5	4	1	4	8409.977	0.010
5	0	5	4	0	4	8624.547	-0.007
5	2	3	4	2	2	9274.587	0.003
6	1	6	5	1	5	10054.360	-0.034
6	0	6	5	0	5	10223.017	-0.018
6	2	5	5	2	4	10677.507	0.019
6	1	5	5	1	4	11155.812	-0.012
7	0	7	6	0	6	11805.239	0.034

Table S09. Measured rotational transitions for the $^{13}C_3$ isotopologue of *Exo-I* conformer of piracetam.

J´	K´a	K´c	J´´	K´´a	K´´c	ν_{obs}	ν_{obs}-ν_{cal}
4	1	4	3	1	3	6764.435	0.043
5	1	5	4	1	4	8426.664	0.043
5	0	5	4	0	4	8642.294	-0.020
5	2	4	4	2	3	8940.109	0.061
5	2	3	4	2	2	9280.411	-0.033
5	1	4	4	1	3	9368.101	-0.021
6	1	6	5	1	5	10074.952	-0.049
6	0	6	5	0	5	10245.585	-0.004
6	2	5	5	2	4	10692.971	-0.009
6	1	5	5	1	4	11169.488	0.023
7	0	7	6	0	6	11832.046	-0.012

Table S10. Measured rotational transitions for the $^{13}C_4$ isotopologue of *Exo-I* conformer of piracetam.

J´	K´a	K´c	J´´	K´´a	K´´c	ν_{obs}	ν_{obs}-ν_{cal}
4	1	4	3	1	3	6811.068	0.041
4	2	2	3	2	1	7419.880	-0.035
5	1	5	4	1	4	8483.641	0.002
5	0	5	4	0	4	8698.246	-0.018
5	2	4	4	2	3	9008.965	0.044
6	0	6	5	0	5	10309.491	-0.019
6	2	5	5	2	4	10773.832	-0.029
6	1	5	5	1	4	11256.771	0.003
7	0	7	6	0	6	11904.874	0.000

Table S11. Measured rotational transitions for the $^{13}C_5$ isotopologue of *Exo-I* conformer of piracetam.

J´	K´a	K´c	J´´	K´´a	K´´c	ν_{obs}	ν_{obs}-ν_{cal}
4	1	4	3	1	3	6816.518	0.057
5	0	5	4	0	4	8704.723	-0.032
5	2	4	4	2	3	9009.285	0.047
5	3	2	4	3	1	9136.324	0.040
5	2	3	4	2	2	9357.510	-0.035
6	1	6	5	1	5	10151.528	-0.018
6	0	6	5	0	5	10319.131	0.005
6	2	5	5	2	4	10775.060	0.004
6	1	5	5	1	4	11252.948	-0.023
7	1	7	6	1	6	11799.390	-0.059
7	0	7	6	0	6	11917.438	0.039

Table S12. Measured rotational transitions for the $^{13}C_6$ isotopologue of *Exo-I* conformer of piracetam.

J´	K´a	K´c	J´´	K´´a	K´´c	ν_{obs}	ν_{obs}-ν_{cal}
4	1	4	3	1	3	6785.396	0.099
4	2	2	3	2	1	7382.072	-0.061
5	1	5	4	1	4	8452.403	0.048
5	2	4	4	2	3	8970.269	0.064
5	2	3	4	2	2	9315.307	0.006
6	1	6	5	1	5	10105.372	-0.036
6	0	6	5	0	5	10275.693	-0.054
6	2	5	5	2	4	10728.627	-0.006
7	1	7	6	1	6	11745.810	-0.061
7	0	7	6	0	6	11866.483	0.038

Table S13. Cartesian coordinates in Angstroms (Å) for the r_s structure of *Exo-I* conformer of piracetam, derived from the ^{13}C isotopologues substitution.

Atom	x	y	z
C	-0.83745	-1.60835	-0.50367
C	-2.44101	-1.1409	-0.34243
C	-2.36535	0.3948	-0.56579
C	-1.08917	0.81331	0.20047
C	0.89433	-0.29891	1.05758
C	1.98449	-0.16259	0.00000
